# Risk perception of COVID-19 among college students in China: Latent profile analysis

**DOI:** 10.3389/fpubh.2022.1041580

**Published:** 2022-11-04

**Authors:** Juanjuan Ren, Zhenxiang Zhang, Yongxia Mei, Wenna Wang, Qianqian Sun, Mingxu Wang, Zhaozhao Hui

**Affiliations:** ^1^School of Nursing and Health, Zhengzhou University, Zhengzhou, China; ^2^College of Public Health, Xi'an Jiaotong University, Xian, China

**Keywords:** college students, COVID-19, risk perception, latent profile analysis, anxiety, depression

## Abstract

**Background:**

The outbreak of the new coronavirus-2019 (COVID-19) has had a significant impact on people's mental and physical health. Meanwhile, people's perceptions of risk may influence their emotional states and preventative behavior during an epidemic. Previous research have revealed the diversity and uniqueness of risk perception, and college students may have a different perspective on risk perception. The objective of this study was to describe the subtypes of risk perception for COVID-19 among college students in China, identify the subtypes' traits, and investigate their affecting variables.

**Methods:**

College students from 10 Chinese provinces participated in a cross-sectional study (*n* = 2,000) that from January 16 to 30, 2022. The latent profiles and influencing factors for risk perception were investigated using latent profile analysis, one-way analysis of variance, and multinomial logistical regression.

**Results:**

The sample group of this survey was 1,946 students, and the response rate was 97.3%. The best model was suggested to consist of three profiles: “neutral risk perception” (20.3%), “perception seriously without susceptible” (52.8%), and “low risk perception” (26.9%). Risk perception of COVID-19 was positively associated with attention to negation information (*r* = 0.372, *p* < 0.01), anxiety (*r* = 0.232, *p* < 0.01), and depression (*r* = 0.241, *p* < 0.01), and negatively associated with perceived social support (*r* = −0.151, *p* < 0.01). Logistic-regressions analyses mainly revealed that the risk perception of three profiles related to having chronic diseases (*OR* = 2.704, *p* < 0.01), medical major (*OR* = 0.595, *p* < 0.01; *OR* = 0.614, *p* < 0.05), without having COVID-19 confirmed cases around (*OR* = 0.539, *p* < 0.01), attention to negative information (*OR* = 1.073, *p* < 0.001; *OR* = 1.092, *p* < 0.001), and perceived social support (*OR* = 0.0.975, *p* < 0.01).

**Conclusions:**

The level of risk perception for COVID-19 among Chinese college students was unsatisfactory, and the risk perception of COVID-19 had significant group characteristics and heterogeneity. Colleges and public health practitioners could have a theoretical and empirical basis to implement risk perception intervention efforts by identifying latent subgroups during the COVID-19 epidemic.

## Introduction

The World Health Organization (WHO) declared the COVID-19 pneumonia epidemic a global public health emergency in 2019 after it was first detected in Wuhan Nationality City, Hubei Province, China. And on March 11, 2020, COVID-19 reached pandemic status throughout the world. Since the outbreak of COVID-19, many colleges have adopted closed management to prevent the spread of the infection among teachers and students (1). However, the COVID-19 epidemic has caused serious threats and heavy losses to health and lives around the world, including Chinese college students (2), and with that comes lots of stress, anxiety, depression, and panic. These negative emotions may trigger serious mental health problems and different perceptions of the epidemic (3). So, these college students' mental health and attitude toward COVID-19 should be taken seriously.

In China, college students are one of the most dynamic groups with strong mobility and socialization. They live a concentrated life, which can easily spread from them to others and lead to serious public health events once infected (4). And, as the special group within COVID-19, they are more vulnerable and suffer greater impact and serious mental health problems. Besides, they are alert and sensitive to health information. Their knowledge, protective behavior, and risk awareness have a significant impact on those around them's risk perception (5, 6). Therefore, college students are the key population for epidemic prevention and control, and it is necessary to focus on their risk perception and mental health.

Risk perception refers to an individual's subjective perception and judgment of various objective external risks (7, 8). Many studies suggested that risk perception was associated with individual preventive behavior, decision-making, and mental health (9–11). Individuals with similar levels of risk perception may adopt different forms of preventive behavior, which is the basis of people's response behavior to public health emergencies (12, 13). In addition, one's risk perception is positively correlated with self-protective behavior during the COVID-19, with self-consciously highly susceptible individuals tending to reduce social contact and increase washing frequency (14, 15). People with higher levels of risk perception are more likely to receive risk warning information more readily and earlier (16, 17). Conversely, people with low risk perceptions are less inclined to adopt protective behaviors and believe they are less likely to be infected, underestimating the severity of diseases (12, 18, 19). From this, it's clear that risk perception is a key factor in individual health behavior and social infectious disease prevention and control and of great significance also.

However, risk perceptions of college students in different countries, including China, are moderate or low in existing studies (3, 10, 20, 21). For example, Soltan et al. (22) defined the score at 75% as high, 50–75% as moderate, and 50% as low level. However, this study found that due to the different number of questions and scoring range of risk perception measurement tools, there are differences in judging the level of risk perception, which leads to the inability to distinguish group differences and analyze the internal characteristics of college students. In addition, a socio-mathematical model of risk perception demonstrates that the heterogeneity of risk perception is manifested by differences in perception by age, gender, expression of feelings, and media consulted in the university community (23). Therefore, the characteristics of risk perception among college students are heterogeneous. When defining the level of risk perception in terms of susceptibility and severity, it will be considered as a whole. However, when in the same group with high levels of risk perception, we do not consider whether individuals are sensitive to severity or susceptibility; some individuals are more dominant in their perception of the severity of the outbreak; and some individuals may be at a disadvantage in perceived severity and more prominent in perceived susceptibility, but we habitually define both as high levels of risk perception, without taking into account possible intra-individual differences. Furthermore, during the COVID-19 epidemic, risk perception was influenced by anxiety, depression, information about the epidemic, gender, age, and the presence of confirmed cases, but it is unclear whether these factors influenced risk perception category characteristics among college students, and further exploration is required (10, 24–26).

Latent profile analysis (LPA) is an individual-centered analysis technique that can improve group category distinction by grouping individuals with similar response patterns into the same subgroup and clarifying response characteristics (27, 28). Therefore, this study aims to investigate the potential categories of the risk perception of COVID-19 among medical students in universities and the differences in their characteristics by using latent profile analysis, which enables us to make reasonable risk assessments, risk regulations, and accurate management decisions according to group characteristics.

This study proposed the following hypotheses: Firstly, according to the socio-mathematical model (23), the heterogeneity of risk perception is manifested by differences in perception by age, gender, expression of feelings, and so on in a university community. Meanwhile, college students were observed to have different levels of knowledge and attitudes toward COVID-19 (29). A survey from China also revealed that income, education, major, and COVID-19 knowledge were the important factors affecting the COVID-19 risk perception of medical college students (4). Therefore, this study hypothesized that college students may perceive varying risks of contracting COVID-19 and that risk perception of COVID-19 may be modified by demographics. Secondly, some studies have discovered that the high COVID-19 risk perception in college students increases their depression and anxiety (30), and risk perception may be affected by perceived social support, and in the high-risk condition, individuals' behavioral intention may be increased by issue salience and deliberate information processing (31, 32). This COVID-19 epidemic has affected college students' mental health and caused post-traumatic stress symptoms, and attention to negative information was a key mediating post-traumatic cognitive factors (33). Besides, the attention bias toward negative information (e.g., news or rumors about COVID-19) increases individuals' tendency to readily percept threatening stimuli or negative information (34). Therefore, this study hypothesized that depression, anxiety, perceived social support, and attention to information are the predictors of risk perception of COVID-19.

## Methods

### Participants

In this survey, participations were voluntary, 16 years old, full-time and internet accessible students, and those who had been diagnosed with anxiety and depression by a psychiatrist previously were excluded from this study.

### Procedure

A cross-sectional survey was conducted from January 16 to 30, 2022, in 10 provinces of China. The anonymous questionnaire was established using an online survey platform (Wen Juan Xing, wjx.cn). We recruited participants by phone, email, and posters, then the questionnaire was forwarded through the Wechat app. Some information, including the purpose of the survey and information confidentiality, was stated at the beginning of the questionnaire to obtain participants' informed consent. Besides, participants were encouraged to share this questionnaire with their friends or classmates, and they were asked to have only one opportunity to fill in this questionnaire. Therefore, snowball sampling was used.

### Measurements

#### Sociodemographic and COVID-19 related data

The demographic information questionnaire included items about age, gender, nationality, place of student source, having chronic diseases, major, and the frequency of COVID-19 related topics discussed with family in the last month, having COVID-19 confirmed cases around, and participating in volunteer activities during COVID-19.

### COVID-19 risk perception scale

The risk perception of COVID-19 was defined as the subjective perception of COVID-19 susceptibility, severity, and controllability (COVID-19 Risk Perception Scale, CRPS). It was measured by nine items on a five-point Likert Scale, assigned 1–5 (1 = strongly disagree, 2 = disagree, 3 = not sure, 4 = agree, and 5 = strongly agree), and total score from 9 to 45 (α = 0.824, split-half reliability = 0.731) (35).

### Attention to positive and negative information scale

In this study, the sub-scale attention to negative information was selected from the Attention to Positive and Negative Information Scale (APNI). It contains 11 items on a five-point likert scale, assigned 1 (very much not in line) to 5 (very much in line), with a total score of 55, and higher scores indicate higher levels of attention to negative information (α = 0.820) (36).

### Perceived social support scale

Perceived social support refers to the extent to which individuals perceive support from various sources of social support and is measured by the Perceived Social Support Scale (PSSS). This comprised various supports, including family support, friend support, and other support, and was measured by 12 items on a seven-point response scale, from 1 to 7, representing “strongly disagree” to “strongly agree.” The scores are summed, and the higher the total score, the higher the level of social support of the individual, and 61–84 are designated as high, 37–60 as moderate, and 12–36 as low level (α = 0.898) (37).

### Generalized anxiety disorder-7

The individual's mental psychological activity in the past 2 weeks measured taken by Generalized Anxiety Disorder-7 (GAD-7). It contains seven items on a four-point Likert Scale, assigned 0 (not at all) to 3 (almost every day), with a total score of 0–21, and 0–5 defined as no symptoms, 6–9 as mild anxiety, 10–14 as moderate anxiety, and 15–21 as severe anxiety, the total score of ≥7 was used as a threshold to screen for anxiety symptoms (α = 0.920) (38– 40).

### Patient health questionnaire-9

The individual's mental and psychological activity in the past 2 weeks was measured by Generalized Anxiety Disorder-7 (GAD-7). It contains seven items on a four-point Likert Scale, assigned 0 (not at all) to 3 (almost every day), with a total score of 0–21, and 0–5 defined as no symptoms, 6–9 as mild anxiety, 10–14 as moderate anxiety, and 15–21 as severe anxiety. The total score of ≥7 was used as a threshold to screen for anxiety symptoms (α = 0.857) (39, 41).

### Statistical analysis

All collected data was entered into Excel V.2019 and transferred to SPSS 26.0 statistical software for analysis. Variables were coded and processed for accuracy and consistency. Using tables and graphs, descriptive statistics were expressed as mean, SD, percentage, and frequency.

The data was then analyzed based on the type of exogenous variable, with nine items from the COVID-19 Scale as observed variables and Mplus 7.4. (i) Log-likelihood (LL), Akaike information criterion (AIC), Bayesian information criterion (BIC), and adjusted Bayesian information criterion (aBIC) were the model fit test indicators. The smaller the above four values are, the better the model fit is. (ii) Entropy, the value range of 01, the closer to 1, the higher the classification accuracy. (iii) when *p* < 0.05, the Lo-Mendell-Rubin and Bootstrapped likelihood ratio test (BLRT) show that the model with K categories outperforms the model with K-1 categories. None of the above indicators had critical values, and the optimal model needed to meet the following criteria: AIC, BIC, and aBIC values were the smallest in the model; Entropy > 0.7 and *p* < 0.05 for LMR and BLRT (42–44).

Furthermore, multinomial logistic regression analysis to identify different category participants' risk perception of COVID-19 and its influencing factors. Pearson correlation analysis was used to explore the association between COVID-19 risk perception and anxiety, depression, perceived social support, and attention to negative information. Moreover, the magnitude of the association between different independent variables with regard to dependent variables was measured using *OR* with a 95% *CI*. All tests were two-sided and α < 0.05 was considered statistically significant.

### Ethical standards

The study was approved by the Biomedical Ethics Committee of the Department of Medicine, Xi'an Jiaotong University (No. 2022-0006), and all procedures performed on Chinese college students were in accordance with the guidelines of the 1964 Helsinki Declaration and its later amendments or comparable ethical standards.

## Results

### Descriptive results

A sample of 1,946 college students participated in this study with a response rate of 97.3%. In Table 1, the mean age of the students was 19.6 (SD 1.7, range 16–30) years. Six hundred fifty-seven (44%) of those who took part were from cities and towns (Table 1).

**Table 1:** College students' sociodemographic and COVID-19 related data characteristics (*N* = 1,946).

**Variable**	**Frequency (%)**
Sex	
Male	649 (33.4)
Female	1,297 (66.6)
Ethnicity	
Han ethnicity	1,745 (89.7)
Others	201 (10.3)
Place of student source	
Cities and towns	857 (44.0)
Rural	1,089 (56.0)
Having chronic diseases	
Yes	93 (4.80)
No	1,853 (95.2)
Major	
Medical	1,707 (87.7)
Non-medical	239 (12.3)
Frequency of COVID19-related topics discussed with family in the last month	
Never	218 (11.2)
At least once a month	550 (28.3)
At least once a week	889 (45.7)
At least once a day	289 (14.9)
Having COVID-19 confirmed cases around	
Yes	17 (0.9)
No	1,793 (92.1)
Unclear	136 (7.0)
Participated in volunteer activities during COVID-19	
Yes	882 (45.3)
No	1,064 (54.7)

### Correlation between risk perception and other variables

As shown in Table 2, the anxiety score of college students was 6.09 ± 5.41 and the depression score was 4.00 ± 4.45. There were 43.73% (851) college students in the state of anxiety symptoms and 30.37% (591) in the state of depression. Besides, Pearson correlation analysis showed that: the risk perception of COVID-19 for college students was positively correlated with attention to negation information (*r* = 0.372, *p* < 0.01), anxiety (*r* = 0.232, *p* < 0.01), and depression (*r* = 0.241, *p* < 0.01). Attention to negation information was positively correlated with anxiety (*r* = 0.556, *p* < 0.01) and depression (*r* = 0.507, *p* < 0.01), and perceived social support was negatively correlated with the risk perception of COVID-19 (*r* = −0.151, *p* < 0.01), attention to negation information (*r* = −0.285, *p* < 0.01), anxiety (*r* = −0.303, *p* < 0.01), and depression (*r* = −0.268, *p* < 0.01), anxiety was highly positively correlated with depression (*r* = 0.802, *p* < 0.01).

**Table 2:** Descriptive statistics and correlations between study variables.

**Variable**	**1**	**2**	**3**	**4**	**5**
The risk perception of COVID-19	1				
Attention to negation information	0.372^**^	1			
Perceived social support	−0.151^**^	−0.285^**^	1		
Anxiety	0.232^**^	0.556^**^	−0.303^**^	1	
Depression	0.241^**^	0.507^**^	−0.268^**^	0.802^**^	1
*Mean*	20.74	30.04	63.36	6.09	4.00
*SD*	7.08	10.01	14.15	5.41	4.45

N = 1,946;

** p < 0.01.

### LPA results

Model fit indices for the LPA analysis are shown in Table 3. Both AIC and BIC values decreased continuously from model one to four as the categories increased, and LMR and BLRT reached significant levels when divided into three categories (both *p* < 0.05) and Entropy > 0.8, but LMR did not reach significant levels when retained to four categories (*p* > 0.05), indicating that model three was superior to model four, which inferred that model three was the best model. Therefore, a three-latent-class model (AIC = 45,485.126, BIC = 45,696.920, and entropy = 0.881) was selected based on its minimal AIC, BIC, and aBIC values with entropy >0.80. Among the three-class solutions, profile 1 described 26.9% and comprised 532 college students; profile 2 described 52.8% and comprised 1,028 college students, and profile 3 described 20.3% and comprised 395 college students (Table 4).

**Table 3:** Fit indices of latent class analysis on risk perception sub-types.

**Model**	**AIC**	**BIC**	**aBIC**	**Entropy**	**LMR**	**BLRT**	**Class probability**
Class 1	52512.291	52612.615	52555.429	–	–	–	1
Class 2	47424.338	47580.397	47491.440	0.870	<0.001	<0.001	0.387/0.613
Class 3	45485.126	45696.920	45576.193	0.881	<0.001	<0.001	0.269/ 0.528/0.203
Class 4	43788.895	44056.424	43903.927	0.970	0.2063	<0.001	0.278/0.194/0.253/0.275

AIC, Akaike information criterion; BIC, Bayesian information criterion; aBIC, adjusted Bayesian Information Criterion; LMR, Lo-Mendell-Rubin likelihood ratio test; BLRT, bootstrapped likelihood ratio test.

**Table 4:** Average latent profile class probabilities for the most likely class membership (row) by latent class (column).

**Latent class^a^**	**Class membership**			
	**1 (*n* = 532)**	**2 (*n* = 1,028)**	**3 (*n* = 395)**	**Total score for risk perception (M ±SD)**
1	0.964	0.036	<0.001	11.48 ± 0.12
2	0.045	0.928	0.026	22.47 ± 0.11
3	<0.001	0.036	0.964	28.53 ± 0.23

a The columns refer to the latent class, and the rows refer to the most likely profit membership. Profile 1 = low risk perception; Profile 2 = perception serious without susceptible; Profile 3 = risk perception neutrals.

**Figure 1:**
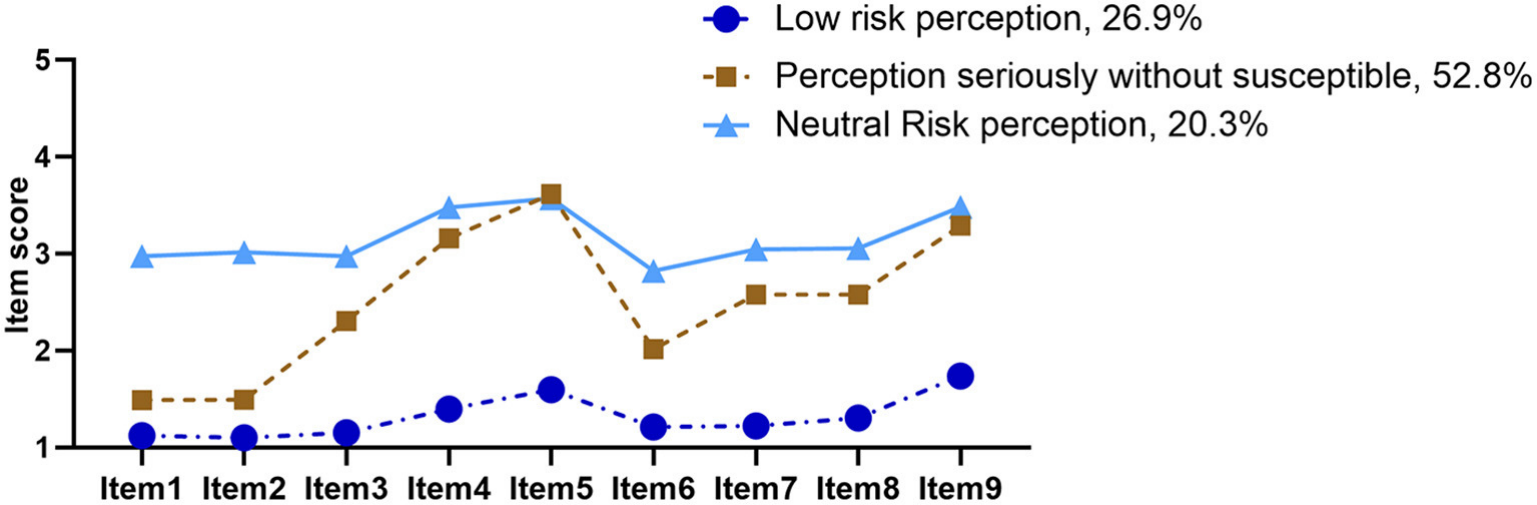
The three profiles of risk perception of COVID-19 by latent profile analysis.

As shown in Figure 1, the first latent profile named “low risk perception” (26.9%) has the lowest scores in perceived risk of COVID-19. The second-and largest latent profile named “perception seriously without susceptible” (52.8%) consisted of those who had high scores in “Once infected, it can have a very serious impact on one's health” and “The COVID-19 is far from over and there is always a risk of infection.” And the third latent profile named “neutral risk perception” (20.3%) consisted of six items tending to choose “not sure.”

### Associations between demographic data and risk perception profiles

The first profile showed that college students who had chronic diseases accounted for the smallest proportion. Overall, there were significant differences in the places of student source, having chronic diseases, major, and having COVID-19 confirmed cases among three latent profiles (both *p* < 0.05). In addition, there were significant differences in the other four variable scores among the three latent profiles (both *p* < 0.05). However, there were no significant differences among the three groups in age, sex, ethnicity, and so on (both *p* > 0.05) (Table 5).

**Table 5:** Demographic information for three profile latent profiles among different college students (*n*, %).

**Variable**	**Low risk perception**	**Perception serious without susceptibile**	**Risk perception neutrals**	***χ^2^*/*F***	***p*-value**
Sex				4.510^b^	0.105
Male	193 (36.9)	324 (31.5)	132 (33.4)		
Female	330 (63.1)	704 (68.5)	263 (66.6)		
Ethnicity				0.487^b^	0.786
Han ethnicity	472 (90.2)	922 (89.7)	351 (88.9)		
Others	51 (9.8)	106 (10.3)	44 (11.1)		
Place of student source				8.118^b^	0.017
Cities and towns	254 (48.6)	448 (43.6)	155 (39.2)		
Rural	269 (51.4)	580 (56.4)	240 (60.8)		
Having chronic diseases				14.006^b^	0.001
Yes	14 (2.7)	47 (4.6)	32 (8.1)		
No	509 (97.3)	981 (95.4)	363 (91.9)		
Major				12.964^b^	0.002
Medical	481 (92.0)	888 (86.4)	338 (85.6)		
Non-medical	42 (8.0)	140 (13.6)	57 (14.4)		
Frequency of COVID19-related topics discussed with family in the last month				10.419^b^	0.109
Never	71 (13.6)	98 (9.5)	49 (12.4)		
At least once a month	143 (27.3)	292 (28.4)	115 (29.1)		
At least once a week	223 (42.6)	483 (47.0)	183 (46.3)		
At least once a day	86 (16.4)	155 (15.1)	48 (12.2)		
Having COVID-19 confirmed cases around				15.107^b^	0.006
Yes	484 (92.5)	964 (93.8)	345 (87.3)		
No	5 (1.0)	7 (0.7)	5 (1.3)		
Unclear	34 (6.5)	57 (5.5)	45 (11.4)		
Participated in volunteer activities during COVID-19				1.220^b^	0.547
Yes	238 (45.5)	456 (44.4)	188 (47.6)		
No	285 (54.5)	572 (55.6)	207 (52.4)		
Anxiety	2.86 ± 4.19	4.05 ± 4.29	5.38 ± 4.78	37.465^c^	<0.001
Depression	4.63 ± 5.45	6.22 ± 5.06	7.66 ± 5.75	37.108^c^	<0.001
Attention to negative information	24.82 ± 11.38	31.25 ± 8.81	33.80 ± 8.13	119.521^c^	<0.001
Perceived social support	66.77 ± 15.67	63.46 ± 13.03	58.57 ± 13.51	39.306^c^	<0.001

χ2 = b; F = c.

A multinomial logistic regression analysis was conducted to identify the relevant factors of risk perception among the three profiles. When the other covariates remained constant, those with chronic diseases (*OR* = 2.704, 95% *CI*: 1.365–5.357) tend to be classified as Profile 3. What's more, medical student (*OR* = 0.614, 95% *CI*: 0.391–0.964), no confirmed cases of COVID-19 around (*OR* = 0.539, 95% *CI*: 0.338–0.859), and perceived social support (*OR* = 0.975, 95% *CI*: 0.965–0.985) was the protective factor for Profile 3, medical student (*OR* = 0.595, 95% *CI*: 0.407–0.869) was also a protective factor for Profile 2, and the lower the attention to negative information (*OR* = 1.073, 95% *CI*: 1.058–1.088; *OR* = 1.092, 95% *CI*: 1.073–1.112) was more likely to belong to Profile 1. Student source location, anxiety, or depression were not related to latent profile memberships (Table 6).

**Table 6:** Multinomial logistic regressions for predicting in three profile latent classes among college students.

**Variable^a^**	**Class 2**		**Class 3**	
	***OR***	**95% *CI***	***OR***	**95% *CI***
Place of student source (rural)	0.890	0.710–1.116	0.801	0.602–1.067
Having chronic diseases (yes)	1.526	0.812–2.867	2.704^**^	1.365–5.357
Major (medical)	0.595^**^	0.407–0.869	0.614^*^	0.391–0.964
Having COVID-19 confirmed cases around (no)	1.188	0.766–1.842	0.539^**^	0.338–0.859
Anxiety	1.003	0.958–1.050	1.046	0.991–1.104
Depression	0.983	0.945–1.022	0.967	0.923–1.014
Attention to negative information	1.073^***^	1.058–1.088	1.092^***^	1.073–1.112
Perceived social support	0.996	0.987–1.004	0.975^***^	0.965–0.985

*** p < 0.001;

** p < 0.01;

* p < 0.05.

a Reference group: Profile 1. OR, Odds ratio; 95% CI, 95% Confidence Interval.

## Discussion

The study was designed to explore the latent profiles of risk perception, and three latent profiles were found ultimately: “low risk perception, perception seriously without susceptible, and neutral risk perception.” The first group, dubbed the “low risk perception group,” included 26.9% of the participants. It indicates that nearly one-third of college students have a low level of risk perception, consistent with the results of previous studies (10). Risk perception refers to people's subjective evaluation and judgment of the severity, characteristics, and management of possible risk exposure. It is influenced by personal, social, cultural, and environmental factors and is based on experience, beliefs, attitudes, and judgments (45). College students have fewer comorbidities and fewer overall health problems and have a different understanding of disease risk than other populations. The lower risk perception of this group may be the result of an interaction of several factors. However, prevention behavior was affected by risk perception (46). People with a low level of risk perception have less possibilities to implement compliance and preventive behavior (18). Perhaps interviews could be conducted with this group to explore the factors that influence risk perception, leading to relevant activities.

Among the participants in profile 2, the “perception seriously without susceptible group” consisted of 52.8% of the participants. The characteristics of this group were high perceived severity, but they were not susceptible to infection under subjective judgment. It is clear that more than half of the students perceived the severity of COVID-19, but their subjective judgment of susceptibility risk may be lower than Han nationality objective levels. Some studies have shown that during the COVID-19 epidemic, social media widely publicizes relevant developments and the internet is also flooded with information about the epidemic (47, 48). Thus, college students, as a highly information-sensitive group (9), have deeply recognized the seriousness of the epidemic and the adverse effects of COVID-19 infection. Besides, on-campus students may consider the campus environment relatively safer compared to off-campus (49). Moreover, Chinese universities mostly adopt closed management and strict prevention and control measures, so college students' daily lives and social mobility are restricted in many ways. The possibility of exposure to infection was reduced, so college students felt that they were less likely to be infected on campus. However, it should be noted that accurate risk assessment and risk perception are crucial; excessive perceived severity can cause unnecessary fear; and self-judgment of not being susceptible can affect effective protective behavior. Hence, universities should adopt a variety of strategies to promote risk communication and two-way interaction among college students.

The third profile, named the “neutral risk perception,” consisted of 20.3% of the participants. They also perceived the seriousness of COVID-19, but were “not sure” on many items. They were unsure whether they would be infected, what effect the COVID-19 infection would have on their bodies, and whether the infection could be cured and controlled. So, it is necessary to know the scientific information about COVID-19 through official media such as Wechat, Facebook, and newspapers (24).

In this study, risk perception was associated with attention to negative information, perceived social support, anxiety, and depression, which was consistent with previous studies (32, 50, 51). The threat stimulus perceived by an individual may lead to attention bias. Attention bias toward negative information increases an individual's tendency to pay attention to threatening stimuli (34). Therefore, the more focused on COVID-19 negative information, the higher perceived threat and risk. People tend to overestimate the risk of negative outcomes due to excessive emotional stress, so ministries of health and education can increase positive messages or news releases, but in moderation; overwhelming messages can also trigger feelings of fear and stress. Besides, the occurrence of risk events can create a stressful environment, generating negative emotions such as anxiety and tension, leading to mental health problems (52). Social support from family and friends may be protective against depressive symptoms because it mediates regulation of risk perception, positive coping (53) and mental health (54). This suggests that during the COVID-19 epidemic, mental health strategies and programs from a risk perception perspective can be designed and integrated into them.

Furthermore, it's found that participants with chronic diseases tend to perceive the severity of the outbreak more easily, which was similar to previous studies (10). Patients with chronic diseases have a higher risk perception and are more likely to be infected after admission, according to data from Portugal (55). This may be related to their poor body resistance and immunity, which could lead to serious complications once infected, making it easier to perceive the risk of infection. On the other hand, risk perception was inevitably influenced by the risk environment in which the participants were exposed (50), chronic diseases may cause psychological stress and stigma for these students, so we should make use of available resources to help them cope with the dual stress of COVID-19 and chronic disease. Additionally, “no confirmed cases of COVID-19 around” and perceived social support were protective factors for Class 3. According to previous literature, predictors of higher risk perception for COVID-19 include the presence of new positive cases in socially exposed populations (25). During the epidemic, campuses are usually in a closed state. Therefore, a serious public health event may occur once someone is infected, which undoubtedly causes great fear and anxiety. However, if there are no suspected or confirmed cases on campus, students will feel relatively safe in the campus environment and be more objective and rational in risk assessments and decision-making.

Medical college students have more rational and reasonable risk perception. According to previous literature (56), medical students have higher levels of knowledge of COVID-19 and preventive behaviors. They are also considering healthier lifestyles in response to the outbreak. However, the level of risk perception of medical students varies at different stages of study and clinical practice (21), and medical students will be the main force in the fight against epidemics in the future, so differentiated education and training are required for students at different grades and practice stages.

In this study, the results show the higher the perceived social support, the lower the risk perception. With the support coming from family, friends, and school, college students have a comprehensive knowledge of COVID-19. Furthermore, social support moderated the relationship between perceived uncontrolled and mental health symptoms (54) and helped buffer the negative emotions associated with high risk perceptions (53). Thus, when students suffer from severe emotional distress that is triggered by excessive risk perception, they should seek psychological or social support, either from a major, family, or friend. In addition, this study also found that the more focused was paid to attention to negative information, the more improbable it belonged to the low risk perception group. Cognitive theories of anxiety propose that selective attention to negative information plays a central role in the development and maintenance of anxiety (57), and young adults attend more to negative emotional information and report more negative emotional reactions to the same information than older adults do (58). Various news reports and negative information were followed during the COVID-19 outbreak, and attention bias to negative information motivated individuals to quickly identify and react to the threat. It may also increase the risk of infection, which can trigger anxiety and depression. Thus, college students should not overly focus on or completely ignore negative information, which can lead to over-or under-assessment of risk. Instead, they should divert their attention appropriately, pay attention to positive information, and enhance their discernment skills.

The results from this study have clinical implications for targeting prevention and intervention for the risk perception of college students. High or low risk perception can lead to risk assessment bias and imbalanced risk decisions, so underestimating or overestimating risk will not be conducive to preventive behavior (6). Risk communication can be seen as the basis for accurate and scientific risk perception that can contribute to the effectiveness of risk management during the COVID-19 outbreak (59). Therefore, after determining which risk perception profile one is in, we can implement these interventions based on the individual's perceived risk characteristics, such as expanding health education on outbreak-related knowledge and focusing on individual preventive behaviors; strengthening social support networks; accurately communicating outbreak risk information through social media; improving the healthcare system during the epidemic to provide precision services to individuals; and involving professionals (such as psychological counselors) in the implementation of scientific and effective risk communication.

This study added to the literature by exploring the potential relationship between personal attention to negative information, perceived social support, anxiety, and depression of college students and their risk perception of COVID-19. And the latent profile analysis conducted found obvious heterogeneity in the risk perception of COVID-19 for college students, which may contribute to developing the appropriate interventions in a targeted manner. We also found obvious heterogeneity in the risk perception of COVID-19 for college students in 10 provinces of China, which could be divided into three latent profile classes. These chronically ill students were studying medicine, and the greater the emphasis on negative information, the greater the perceived severity.

However, this study also has some limitations. The sample size is small and cannot represent the risk perception characteristics of Chinese college students in all provinces. And, during this survey, the outbreak was occurring in some areas (especially in Shanxi and Henan), so some students could not go home, which may have led to perceptual biases in risk perceptions and somewhat affected the credibility of the results. Besides, all questionnaires were self-reported, which may lead to bias in results without longitudinal tracking of time-varying patterns of risk perception. A number of researchers have studied risk perception in Chinese college students during the COVID-19 outbreak. In addition, the current study is the first effort to identify subtypes of risk perception among the college population using latent profile analysis to designate categories. The results of this study have implications for the development of targeted risk communication interventions for subtypes.

## Conclusion

In general, risk perception is influenced by emotion, attention, society, environment, and other factors, and an individual's risk perception of COVID-19 has significant group characteristics and heterogeneity. This study has identified three profiles of risk perceptions toward COVID-19 for a sample of Chinese college students, which indicates that an accurate perception of this pandemic risk is beneficial to psychological health and preventive behavior during the outbreak of COVID-19. These results may provide a theoretical and empirical basis for colleges and public health practitioners to implement risk perception intervention efforts during the COVID-19 epidemic.

## Data availability statement

The original contributions presented in the study are included in the article/supplementary material, further inquiries can be directed to the corresponding author.

## Author contributions

Material preparation, data collection, and analysis were performed by JR, ZZ, YM, WW, QS, MW, and ZH. The first draft of the manuscript was written by JR, YM, and WW. All authors contributed to the study conception and design, commented on previous versions of the manuscript, read, and approved the final manuscript.

## Funding

This research was supported by the National Social Science Foundation (Grant No: 21VSZ084) and the Medical Education Program of Henan Province (Grant No: Wjlx2021016). The funders had no role in this study design, data collection and analysis, decision to publish, or preparation of the manuscript.

## Conflict of interest

The authors declare that the research was conducted in the absence of any commercial or financial relationships that could be construed as a potential conflict of interest.

## Publisher's note

All claims expressed in this article are solely those of the authors and do not necessarily represent those of their affiliated organizations, or those of the publisher, the editors and the reviewers. Any product that may be evaluated in this article, or claim that may be made by its manufacturer, is not guaranteed or endorsed by the publisher.
